# Functional Characterization of *PsnNAC036* under Salinity and High Temperature Stresses

**DOI:** 10.3390/ijms22052656

**Published:** 2021-03-06

**Authors:** Xuemei Zhang, Zihan Cheng, Wenjing Yao, Kai Zhao, Xueyi Wang, Tingbo Jiang

**Affiliations:** 1 State Key Laboratory of Tree Genetics and Breeding, Northeast Forestry University, Harbin 150040, China; zhangxuemei199111@gmail.com (X.Z.); zxm_19910906@sina.com (Z.C.); yaowenjing@njfu.edu.cn (W.Y.); zhangdi_2002@sina.com (K.Z.); wxy20211029@163.com (X.W.); 2 Co-Innovation Center for Sustainable Forestry in Southern China/Bamboo Research Institute, Nanjing Forestry University, Nanjing 210037, China

**Keywords:** *Populus simonii × P. nigra*, *PsnNAC036*, transcription factor, salt stress, HT tolerance

## Abstract

Plant growth and development are challenged by biotic and abiotic stresses including salinity and heat stresses. For *Populus simonii × P. nigra* as an important greening and economic tree species in China, increasing soil salinization and global warming have become major environmental challenges. We aim to unravel the molecular mechanisms underlying tree tolerance to salt stress and high temprerature (HT) stress conditions. Transcriptomics revealed that a *PsnNAC036* transcription factor (TF) was significantly induced by salt stress in *P. simonii × P. nigra*. This study focuses on addressing the biological functions of *PsnNAC036*. The gene was cloned, and its temporal and spatial expression was analyzed under different stresses. *PsnNAC036* was significantly upregulated under 150 mM NaCl and 37 °C for 12 h. The result is consistent with the presence of stress responsive *cis*-elements in the *PsnNAC036* promoter. Subcellular localization analysis showed that *PsnNAC036* was targeted to the nucleus. Additionally, *PsnNAC036* was highly expressed in the leaves and roots. To investigate the core activation region of *PsnNAC036* protein and its potential regulatory factors and targets, we conducted trans-activation analysis and the result indicates that the C-terminal region of 191–343 amino acids of the *PsnNAC036* was a potent activation domain. Furthermore, overexpression of *PsnNAC036* stimulated plant growth and enhanced salinity and HT tolerance. Moreover, 14 stress-related genes upregulated in the transgenic plants under high salt and HT conditions may be potential targets of the *PsnNAC036*. All the results demonstrate that *PsnNAC036* plays an important role in salt and HT stress tolerance.

## 1. Introduction

Various abiotic stresses such assoil salinity, drought, extreme temperature, and heavy metals affect plant growth, development, and productivity [1]. Many important economic trees are particularly sensitive to these environmental stresses, such as *Populus. simonii × P. nigra*. *P. simonii × P. nigra* is an endemic plant found in the Yellow River basin and the northern part of mainland in China [2]. It has been used as an urban afforestation tree species in northeast and northwest China. Importantly, the wood is widely used for paper, fiber, matchsticks, and building materials [3]. In recent years, the influence of adverse environmental conditions on the growth of *P. simonii × P. nigra* has become more and more severe. Therefore, it is urgent to improve the stress tolerance of this important wood plant through molecular genetics and breeding.

The NAC (NAM, ATAF, and CUC) family is one of the most important transcription factor (TF) gene families in plants. Most NAC TFs are reported to participate in regulation of plant growth and developmental processes [4], including shoot apical meristem formation [5], seed and embryo development [6], lateral root development [7], cell division [8], and leaf senescence [9]. Besides development, NAC TFs also play vital roles in plant response to abiotic stresses, such as salinity, heat, cold, and drought [10]. There are 138 NAC TFs in *Arabidopsis thaliana*, 289 in *Populus trichocarpa*, and 42 in *Nicotiana tabacum* according to PlantTFDB (http://planttfdb.gao-lab.org/family.php?fam=NAC accessed on 4 March 2021). Regarding Arabidopsis, there are a large number of studies of NAC TFs in stress responses. For example, *ANAC019*, *ANAC055*, and *ANAC072* were induced by drought, salinity, and abscisic acid (ABA), and they play a vital role in ABA signaling and osmotic stress [11]. *ANAC078* was confirmed to positively regulate anthocyanin biosynthesis under high light [12]. *ANAC002* (*ATAF1*) can be induced by drought, salinity, ABA, methyl jasmonate, and wounding. Overexpression of the *ATAF1* led to increased Arabidopsis sensitivity to ABA, salt and oxidative stresses [13]. In recent years, NAC TFs have been characterized in poplar to be associated with a stress response. For example, *NAC13* was significantly induced in the roots of 84K poplar by salt stress, and *NAC13* overexpression enhanced poplar salt tolerance [14]. In addition, *PeNAC036* in *P. euphratica* was strongly induced by drought, ABA, and salt, and played a positive role in abiotic stress responses [15]. Transgenic Arabidopsis overexpressing a poplar *NAC57* gene displayed higher seed germination, superoxide dismutase, and peroxidase activities under salt stress than wild type (WT) plants [16]. Therefore, studies of poplar NAC TFs are important to understand the molecular mechanisms of stress response and how to enhance stress tolerance in woody plants.

NAC proteins contain a highly conserved NAM domain at the N-terminus, which can be divided into five subdomains known as A-E, and a highly divergent activation domain at the C-terminus [17]. Based on the conserved N-terminal NAM domain, the NAC family was divided into 18 subfamilies from NAC-a to r in populous [10]. The *PsnNAC036* in this study belongs to the NAC-d subfamily. It was cloned and its relative expression levels were analyzed under various treatments. A total of eight transgenic tobacco lines overexpressing the TF were obtained. Morphological and biochemical measurements indicate that *PsnNAC036* can enhance salinity and HT tolerance of transgenic tobacco plants. RT-qPCR results showed that overexpression of *PsnNAC036* upregulated the expression of 14 stress-related genes in tobacco. These results demonstrate that *PsnNAC036* plays an important role in improving plant salinity and HT tolerance.

## 2. Results

### 2.1. Transcript Analysis of 289 NAC TF Genes in Populus

**Figure 1:**
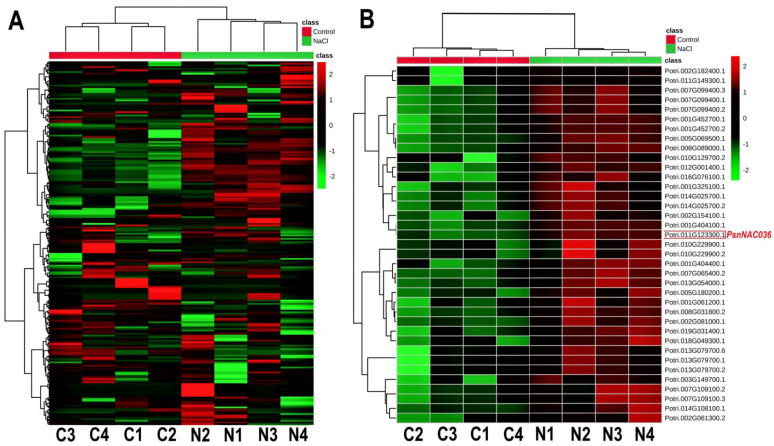
Heatmaps of the relative expression of *NAC* genes in control and NaCl treated *P. simonii × P. nigra* seedlings. (**A**) Relative expression patterns of 289 *NACs* in salt stress versus control. (**B**) Heatmap of the 37 differentially expressed *NAC*s. Red and green colors indicate high and low expression, respectively. The colored scale bar represents fold changes of transcription levels betweenNaCl stress and control. C1, C2, C3, and C4 represent the four biological replicates under control conditions; N1, N2, N3, and N4 represent the four biological replicates under NaCl stress.

To analyze changes of expression levels of the NAC TFs, the fragment per kilobases per million reads (FPKM) of 289 NAC transcription factor genes from *Populus simonii × P. nigra* leaves were retrieved from an RNA-seq dataset [18], which has been deposited in NCBI SRA (accession SRP267437). The salt-stress responsive NAC TFs were visualized using a heatmap (Figure 1A). Based on a fold change of more than 1.2 and a false discovery rate (FDR) smaller than 0.05, 37 differentially expressed NACs were identified (Figure 1B). Among these genes, *PsnNAC036* was significantly upregulated after salt stress treatment of the *P. simonii × P. nigra* seedlings compared to control.

### 2.2. Bioinformatics and Gene Expression Analysis of the PsnNAC036 Gene

To analyze the cDNA and encoded amino acids sequences of *PsnNAC036*, we isolated *PsnNAC036* gene from *P. simonii × P. nigra*. The results showed that the coding sequence of the *PsnNAC036* is 1029 bp in length (Supplementary Figure S1A), and encodes a 343 aa protein with 16.2% alpha helix, 15.8% extended strand, 4.0% beta turn, and 64.0% random coil. Five highly homologous genes were found in the NCBI database according to amino acid sequence blast, including *PtrNAC036* from *Populus trichocarpa*, XP_011029436 from *Populus euphratica* which has 155 NAC TFs, *PtrNAC044* from *Populus trichocarpa*, *ANAC072* (AT4G27410.2) from *A. thaliana* and *Nta009260* from *Nicotiana tabacum* (Supplementary Figure S1B). They shared 98.5, 97.1, 79.4, 70.5, and 62.0% amino acid sequence similarity with the *PsnNAC036*, respectively (Supplementary Figure S1C). According to the sequence alignment, these NACs all contain five highly conserved motifs MA, MB, MC, MD, and ME, which constitute a NAM domain at the N-terminus. Among them, MC and MD were known to bind to DNA [19]. In addition, they all have a predicted nuclear localization signal (NLS) region between 119–150 amino acids (Supplementary Figure S1C).

**Figure 2:**
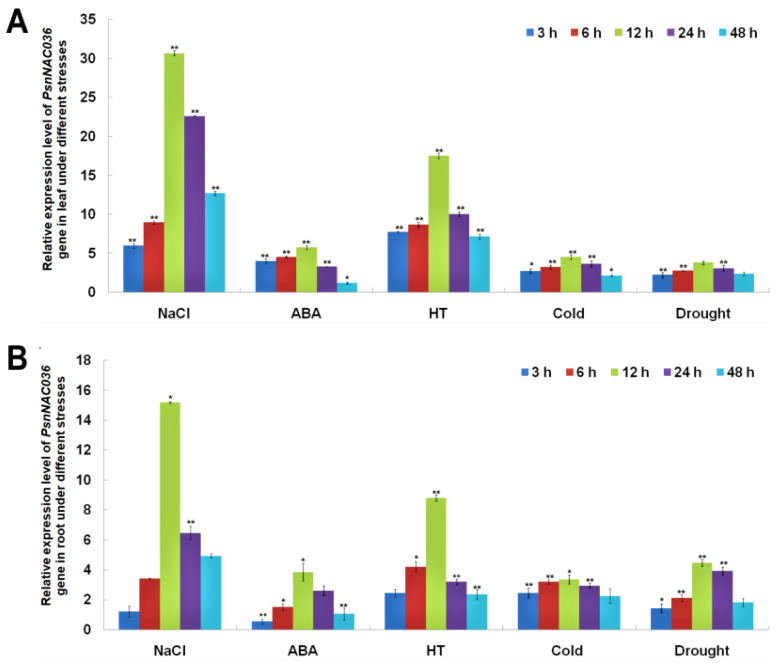
Expression analysis of the *PsnNAC036* under various treatments. Relative expression levels of *PsnNAC036* in leaves (**A**) and roots (**B**) after NaCl (150 mM), ABA (50 µM), high temperature (HT, 37 °C), cold (4 °C), or drought treatments. Expression levels in the control samples were normalized to 1. The data are from three independent experiments. Student’s *t*-test: *t*: * *p* < 0.05; ** *p* < 0.01. Error bars indicate mean ± standard deviation.

To investigate temporal expression patterns of *PsnNAC036* under different stress conditions, we treated the *P. simonii × P. nigra* seedlings with NaCl, ABA, HT, cold, or drought, and harvested the leaves and roots at 0, 3, 6, 12, 24, and 48 h, respectively. The RT-qPCR results showed that the relative expression level of *PsnNAC036* was upregulated in the leaves after different treatments, and it peaked at 12 h. In particular, the gene expression was significantly upregulated after salinity and HT treatments. The *PsnNAC036* expression showed similar expression patterns in the root tissues and the relative expression level of the gene in the root reached 15.2 and 8.8 times higher under NaCl and HT at 12 h than control, respectively (Figure 2).

### 2.3. Characterization of the PsnNAC036 Promoter Sequence

**Figure 3:**
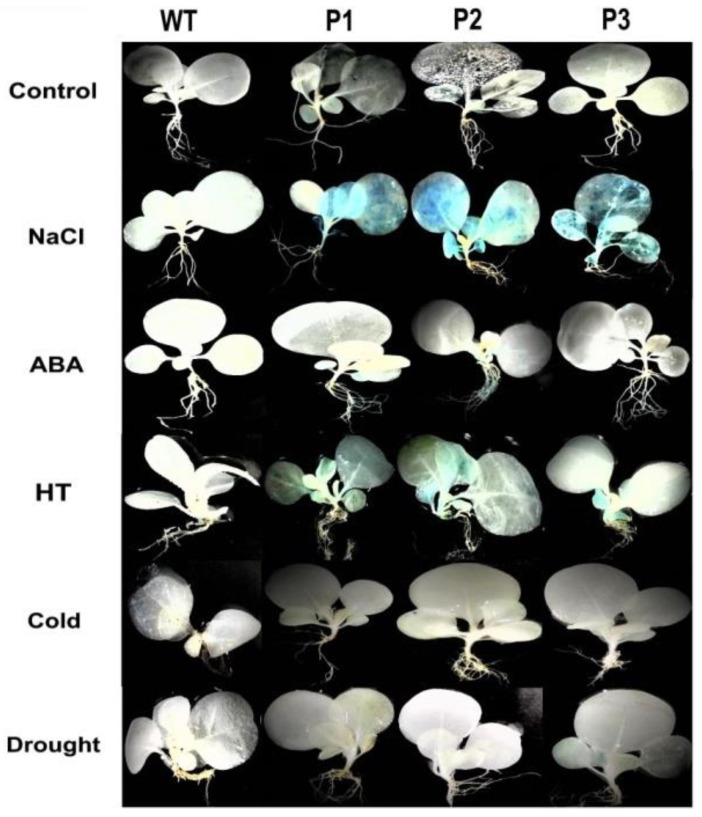
*GUS* activity analysis of the *PsnNAC036* promoter under different treatments.

Promoters contain different transcriptional regulatory elements that can be recognized and bound by RNA polymerases and transcription factors. Some of the regulatory elements play important roles in response to external stimuli [20]. To explore the structure of *PsnNAC036* promoter, the promoter sequence of *PsnNAC036* (from −1 to −1726 bp) in length was isolated from the *P. simonii × P. nigra*. Different *cis*-elements in the promoter sequence were predicted (Supplementary Figure S2), including ABRE, Box 4, CGTCA-motif, G-box, I-box, W-box, MYC, O_2_-site, etc. by PlantCARE (Supplementary Table S1). Then the promoter was cloned into the pBI121 vector to drive the *GUS* gene expression. To further investigate the promoter activity, we obtained stable transgenic tobacco lines expressing *GUS* under the control of the *PsnNAC036* promoter. *GUS* histochemical staining showed that only young leaves of transgenic tobacco showed a light blue color (Figure 3). The same pattern was observed under cold and drought stresses. After ABA treatment, the *GUS* activity was mainly expressed in the roots, while after 150 mM NaCl treatment for 12 h the *GUS* activity was found throughout the seedlings, especially in the leaves. A similar phenomenon was observed under HT stress, in spite of lower staining intensity than with NaCl treatment. The results confirmed that the *PsnNAC036* gene was responsive to NaCl and HT stresses.

### 2.4. PsnNAC036 Protein Is Localized to the Nucleus and Potent Activation Domain in C-terminal Domain

**Figure 4:**
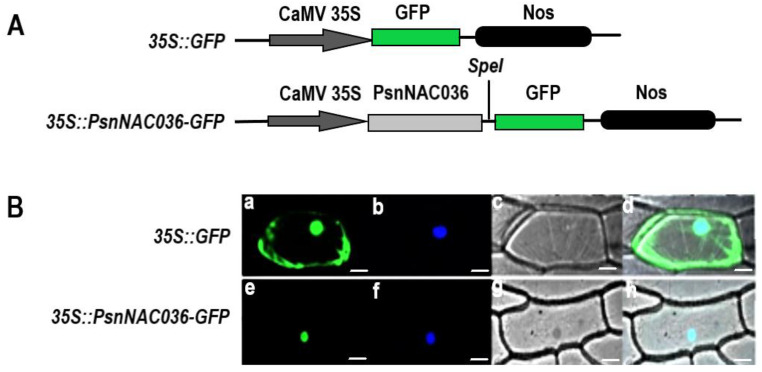
Subcellular localization of the *PsnNAC036* protein. (**A**) Schematic map of the T-DNA inserted in the *35S::GFP* binary vector. (**B**) The *35S::PsnNAC036-GFP* fusion construct and the positive control *35S::GFP* plasmid were introduced into onion epidermal cells by particle bombardment. GFP fluorescence was observed by confocal laser scanning microscopy. (**a**,**e**) are fluorescence images observed in a dark field (green); (**b**,**f**) are 2-(4-Amidinophenyl)-6-indolecarbamidine dihydrochloride (DAPI) staining, which is specific for the nucleus (blue); (**c**,**g**) are light images observed in bright field; (**d**,**h**) are merged images of dark field and bright field. Scale bar = 20 μm.

To determine the predicted nuclear localization of the *PsnNAC036* protein (http://cello.life.nctu.edu.tw/cello2go/ accessed on 4 March 2021), the *PsnNAC036* ORF without the termination codon was fused with GFP (Figure 4A). *35S::PsnNAC036-GFP* and positive control *35S::GFP* were transformed into onion epidermal cells by particle bombardment, respectively. As shown in Figure 4B, the fluorescent signal of *PsnNAC036-GFP* was only found in the nucleus; however, the fluorescent signal of the GFP control was distributed throughout the cell. The results clearly showed that the *PsnNAC036* protein was indeed targeted to the nucleus.

**Figure 5:**
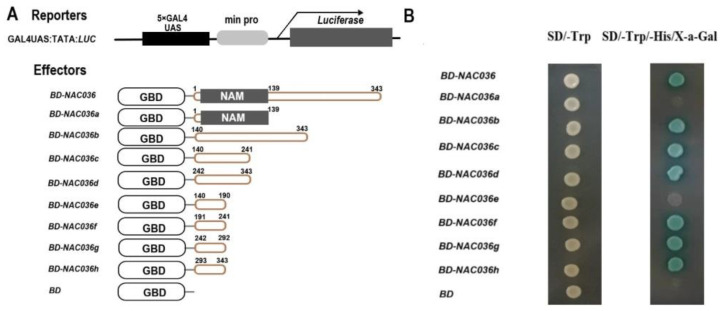
Transactivation analysis of *PsnNAC036* protein. Transactivation analysis of *PsnNAC036* protein. (**A**) Schematic diagrams of the effector and reporter constructs. The reporter construct includes GAL4 binding sites UAS and minimal CaMV35S promoter (min pro) upstream from the luciferase reporter gene. The effector constructs including *BD-NAC036* and GAL4-BD were fused with full-length *PsnNAC036*; *BD-NAC036a*, N-terminal domain (1–139 aa); *BD-NAC036b*, C-terminal domain (140–343 aa); *BD-NAC036c*, half of *BD-NAC036b* (140–241 aa); *BD-NAC036d*, 242–343 aa; *BD-NAC036e*, half of *BD-NAC036c* (140–190 aa); *BD-NAC036f*, 191–241 aa; *BD-NAC036g*, half of *BD-NAC036d* (242–292 aa); and *BD-NAC036h*, 51 aa of C-terminal domain (293–343 aa). BD was the negative control for pGBKT7. (**B**) Yeast assay.

To test the transcriptional activity of *PsnNAC036*, the full length ORF of the *PsnNAC036* sequence was inserted into the pGBKT7 vector.As shown in the transactivation result (Figure 5A), *PsnNAC036* clearly acted as a TF. To determine the functional domains required for activating transcription, we used a yeast assay system to test the activation domains of *PsnNAC036*. Deletion analysis indicates that the constructs including fragments with amino acids 191–241, 242–292, or 293–343 resulted in transactivation, strongly suggesting that the entire region of 191–343 possesses transactivation activity (Figure 5B).

### 2.5. PsnNAC036 Enhanced Tolerance to Salt and HT Stresses in Transgenic Tobacco

**Figure 6:**
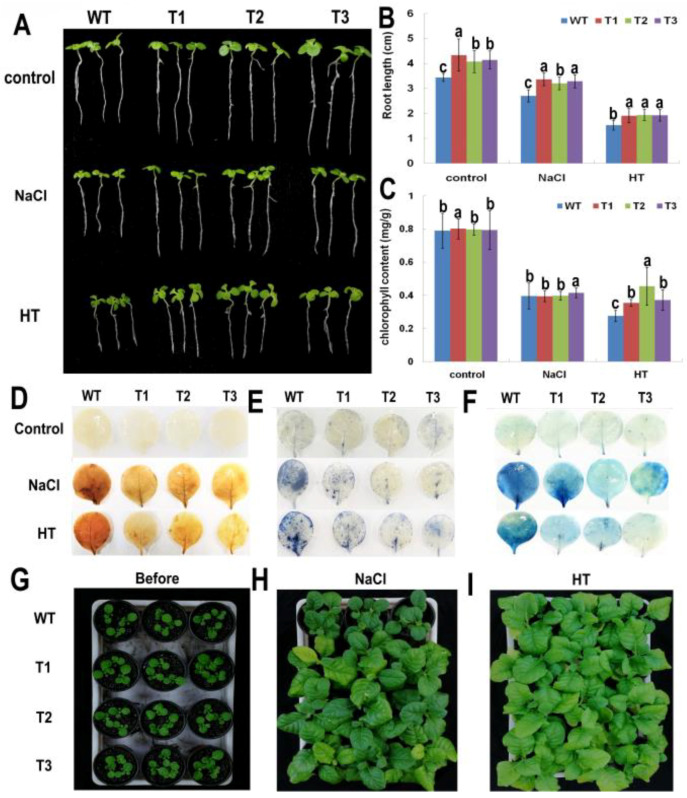
Morphological analysis and histochemical staining of transgenic tobacco lines under NaCl and HT stresses. (**A**) Growth of *PsnNAC036* transgenic lines T1, T2, and T3 seedlings in comparison with WT under control, 150 mM NaCl and 37 °C high temperature (HT) conditions. (**B**) Root length assay of the transgenic lines, compared to WT under control, 150 mM NaCl and HT conditions. (**C**) Chlorophyll content of transgenic tobacco lines and WT plants. The statistical analysis was done using a one-way analysis of variance (ANOVA) with a post-hoc with Tukey lines with an alpha value of 0.05. Different letters indicate significant difference between sites. (**D**) 3,3′-Diaminobenzidine (DAB) staining; (**E**) Nitroblue tertazolium (NBT) staining; (**F**) Evans blue staining; (**G**) Four-week-old tobacco plants in soil right before treatments; (**H**) 200 mM NaCl treatment for two weeks; (**I**) HT treatment for two weeks.

Transgenic tobacco lines overexpressing the *PsnNAC036* (Figure 6A) and WT were screened for the presence of transgenes by gDNA PCR with the gene specific primers F1 (5′-ATGGGACTGCAAGAAACAGACC-3′) and R1 (5′-TCACTGCCTAAACCCATACCCA-3′) and RT-PCR with primers F2 (5′- CTTGAATCCTCTCGCAAAAGTG-3′) and R2 (5′-GAGCCGGTCATCAATCTCTGTC-3′). The housekeeping gene *actin* (aF: 5′-GCTTGCTTACATTGCTCTCGAC-3′, aR: 5′- TGCTTCCGGCTCTGATGTTGTG-3′) for RT-PCR was designed from internal actin gene (GenBank accession number: X69885). As shown in Supplementary Figure S3, *PsnNAC036* fragments were amplified in the transgenic tobacco lines and had the same length as the positive control, but were not detected in the non-transgenic WT.

To investigate the functions of the *PsnNAC036*, transgenic lines T1, T2, T3, and WT were grown in MS, MS with 150 mM NaCl, and MS under 37 °C for two weeks. Under the control condition the plants all grew well. However, the root length of the transgenic lines was about 1.12 ± 0.07 times longer than WT. Under high NaCl or HT treatments, the growth of WT was obviously affected, and the size of WT was smaller than the transgenic lines (Figure 6A). Additionally, the root length of transgenic lines was approximately 1.22 ± 0.03 and 1.26 ± 0.01 times longer than WT under NaCl and HT treatments, respectively (Figure 6B). Besides, after salt treatment, the root length ratios of WT, T1, T2, and T3 (to the transgenic lines) were 72.29%, 77.58%, 78.61%, and 79.40%, respectively, compared to the control plants. Among them, T1 and T3 were more significant than WT. After HT treatment, the root length ratios of WT, T1, T2, and T3 (to the transgenic lines) were 40.95%, 43.95%, 47.53%, and 46.50%, respectively, compared to the control plants. These results demonstrate that the transgenic tobacco plants overexpressing *PsnNAC036* exhibited significant NaCl and HT tolerance.

### 2.6. Changes in Leaf Chlorophyll Content and Physiological Indexes

Leaf chlorophyll is central for light capture and energy exchange between the biosphere and the atmosphere [21], and it serves as a physiological index closely related to plant metabolism and stress resistance [22]. To detect the changes of leaf chlorophyll content and physiological indexes in different tobacco lines, chlorophyll a and chlorophyll b were extracted and quantified from leaves of the transgenic lines and WT plants after salt and HT treatments.The results of chlorophyll content from T1, T2, and T3 lines compared with WT showed that there is no significant difference under normal and high salt conditions, while after HT treatment, the total chlorophyll content of transgenic tobacco lines was 1.29–1.65 fold higher than that of the WT plants (Figure 6C). The results of physiological parameters showed that under normal conditions, the activities of superoxide dismutase (SOD) and peroxidase (POD), as well as proline content were nearly the same in the transgenic lines and WT. However, under salt treatment, SOD and POD activities and proline content were all significantly higher in transgenic lines than in WT. MDA contents of WT were also approximately 1.2- and 1.6-times larger than those of the transgenic plants under normal and salt stress conditions, respectively (Supplementary Figure S4).

### 2.7. Histochemical Staining and Growth Assay of the PsnNAC036 Transgenic Plants

Histochemical staining including diaminobenzidine (DAB), nitroblue tetrazolium (NBT) and Evans blue was carried out to investigate the degree of oxidative damage and cell death. The transgenic and WT plants were treated with NaCl and HT for 12 h. Under control conditions, there was no significant difference between the transgenic and WT leaves. However, under NaCl and HT conditions, intense coloring was observed in the WT leaves after NBT, DAB, and Evans blue staining, in contrast to light staining in the transgenic lines (Figure 6D–F). The results showed that accumulation of reactive oxygen species (ROS) and cell death in the transgenic plants were greatly reduced compared to the WT NaCl and HT treatments.

Under normal growth conditions, the WT and *PsnNAC036* transgenic lines grew similarly. However, after the one-month-old seedlings were treated with 200 mM NaCl or kept at 37 °C for two weeks, the growth of WT was severely retarded, compared to transgenic lines, especially after high salt treatment (Figure 6G–I).

### 2.8. PsnNAC036 Alters the Expression of Stress-Related Genes

**Figure 7:**
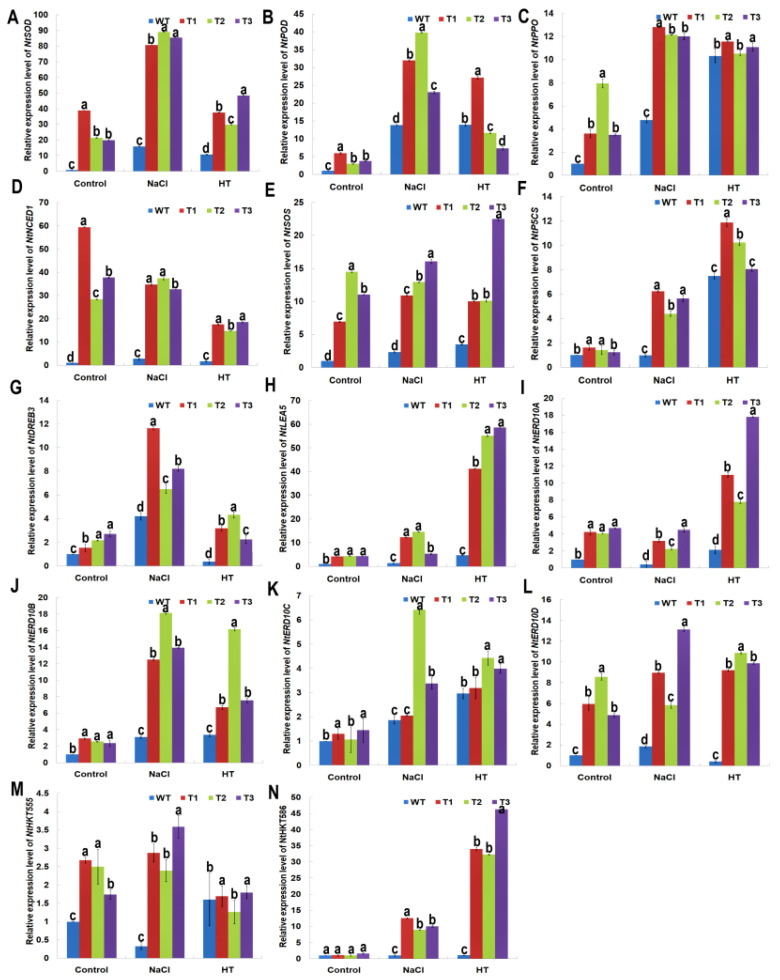
Genes expression profiling of stress-responsive genes in the *PsnNAC036* transgenic plants under NaCl and HT treatments. The relative fold change in expression of (**A**) *NtSOD*; (**B**) *NtPOD*; (**C**) *NtPPO*; (**D**) *NtNCED1*; (**E**) *NtSOS*; (**F**) *NtP5CS*; (**G**) *NtDREB3*; (**H**) *NtLEA5*; (**I**) *NtERD10A*; (**J**) *NtERD10B*; (**K**) *NtERD10C*; (**L**) *NtERD10D*; (**M**) *NtHKT555*; (**N**) *NtHKT586* genes in the *PsnNAC036* transgenic lines under control, high salt and heat conditions. The statistical analysis was done using a one-way ANOVA with a post-hoc with Tukey lines with an alpha value of 0.05. Different letters indicate significant difference between sites.

To elucidate molecular functions of the *PsnNAC036*, relative expression levels of 14 genes related to stress response in the transgenic plants were analyzed after NaCl and HT treatments by RT-qPCR. As shown in Figure 7, these genes were significantly upregulated in the transgenic plants compared to WT under control and stress treatments. Expression of *NtSOD* that conferred osmotic stress tolerance was found to increase by about 26.8 folds in transgenic lines under control conditions, 85.1-fold under salt stress, and 38.7-fold under HT stress, compared to the WT. The relative expression level of *NtPOD* was upregulated by 4.2-fold, 31.6-fold, and 15.4-fold compared to the WT under control, salinity, and HT, respectively. Furthermore, *NtP5CS* and *NtLEA5* involved in osmotic adjustment and membrane protection [23] were also upregulated by 1.4- and 4.3-fold in the transgenic tobacco, compared to WT, respectively (Figure 7F,H). Under high salt stress, their expression levels were upregulated by 5.4- and 10.8-fold, respectively. Under HT, their expression was significantly upregulated by 10.1- and 51.7-fold, respectively. *NtPPO* encodes a polyphenol oxidase involved in plant stress tolerance. Under normal conditions, the transcript of *NtPPO* in transgenic lines was upregulated by 5.1-fold, while under NaCl and HT, it was upregulated by 2.6- and 1.2-fold, respectively, compared to WT. Moreover, the transcript levels of *NtERD10A*, *NtERD10B*, *NtERD10C*, and *NtERD10D* [24] also showed higher expression (4.4-, 2.6-, 1.3-, and 6.5-fold, respectively) in transgenic plants compared to the WT (Figure 7H,I). The fold-change values were 3.3, 14.9, 4.0, and 9.3 under salt stress, and 12.2, 10.2, 3.9, and 10.0 under HT treatment, respectively. Also, *NtHKT555*, *NtHKT586*, and *NtSOS* encode Na^+^/H^+^ antiporters [25], and their expression levels were all higher in the transgenic lines than in the WT under control or treatments. Interestingly, *NtNCED1,* involved in the biosynthesis of SA, JA, and ABA [26], was significantly upregulated (41.9, 35.0, and 17.0 fold in the transgenic lines under control, NaCl and HT conditions, respectively). The upregulation of these stress responsive genes may account for the NaCl and HT tolerance of the transgenic plants.

## 3. Discussions

With the intensive farming and climate change, salinity and heat have become serious environmental challenges that reduce agricultural productivity of many crops world-wide. Salinity causes osmotic stress and cellular toxicity to plants [27], and heatstress often leads to protein misfolding and degradation that will affect critical cellular reactions and processes [28]. In this study, we found that salt and heat stresses can negatively affect the growth of *P. simonii × P. nigra* (Supplementary Figure S5). It is therefore intriguing to investigate molecular mechanisms underlying the stress responses of this woody plant.

The NAC family first identified in Petunia is one of the largest plant-specific TF families [29]. Many NAC family members have been shown to play important roles in gene regulation under environmental stresses [30,31,32,33]. In the reference plant Arabidopsis, many studies have elucidated the functions of NAC TFs in plant response to various abiotic stresses. For example, *ANAC019* functions as an upstream regulator of several drought-induced genes such as *DREB2A*, *DREB2B*, *ARF2*, *MYB21*, *MYB24*, and thereby plays an important role in stress response and floral development [34]. NAC TFJUNGBRUNNEN1 (*ANAC042*) was found to respond to H_2_O_2_ treatment and enhance plant thermo-tolerance [35]. In addition, overexpression of *ANAC069* can decrease plant ROS scavenging capability and proline biosynthesis, while knock down of *ANAC069* improves plant salt and osmotic stress tolerance [36], indicating *ANAC069* as a negative regulator of plant stress tolerance. In Arabidopsis, the homologous gene of *PsnNAC036* is *ANAC07*2 (AT4G27410.2, *RD26*), which has been reported to respond to drought, salinity, ABA, SA, and MeJA [37,38]. In this study, we provide several lines of evidence, showing that the *PsnNAC036* can improve plant salinity and heattolerance. Besides, the results of RT-qPCR of seedlings under ABA, cold and drought stress treatments also showed that *PsnNAC036* expression can be induced by these abiotic stresses. Further studies are needed to explore the *PsnNAC036* gene response to other abiotic stresses and determine whether it may have similar functions to the Arabidopsis *ANAC072* homolog.

Plant promoter includes a conservative basic core promoter region and upstream *cis*-acting elements that are crucial to the specificity and activity of gene transcription [39]. In this study, promoter region of *PsnNAC036* gene which is 1726 bp isolated. According to the analysis of PlantCARE software, the promoter contains a variety of *cis*-elements. Most of these elements are related to stress response, such as as-1 belongs to oxidative stress-responsive element, DRE core is involved in dehydration response, and MYC is related to chilling response. Treating *PsnNAC036* promoter transgenic tobacco with salt, cold, heat, ABA, and drought, we found that the *GUS* activity is mainly expressed under salt and HT treatment. This suggested that there may exist other elements in the *PsnNAC036* promoter that contribute to respond high salt and HT stress.

Production of ROS including the superoxide anion (O_2_^−^), hydrogen peroxide (H_2_O_2_), and hydroxyl radicals (OH^−^) are associated with plant salt stress responses [40]. MDA is a cellular indicator of lipid peroxidation when plants experience oxidative stress caused by environmental challenges, such as salinity and heat [41]. To maintain the redox homeostasis, plants regulate antioxidant enzymes and molecules [42]. SOD and POD are key antioxidant enzymes in ROS-scavenging [43]. In addition, salt stress also causes osmotic stress.Proline is known to function as an important osmolyte in adjusting cellular osmotic balance [44]. In this study, we measured several physiological parameters including SOD, POD activity, proline, and MDA contents in *PsnNAC036* transgenic plants and WT under salt treatment, and found that SOD, POD, and proline content in transgenic lines were increased after stress treatment and MDA content was lower when compared to WT. These results demonstrate that *PsnNAC036* can improve the salt tolerance by regulating the redox and osmotic processes. Also, since DAB, NBT, and Evans blue are dyes commonly used to detect ROS levels and cell death in plants [45,46], the staining results clearly show that overexpression of *PsnNAC036* reduced ROS accumulation and cell death in plants (Figure 7). Our findings are consistent with previous reports that NAC proteins play a crucial role in mediating gene regulation of the anti-oxidative system under stress conditions, thereby conferring higher stress tolerance [47].

Salinity depresses plant growth through lowering water content and excessive accumulation of predominant ions including sodium (Na^+^), chloride (Cl^−^), calcium (Ca^2+^), potassium (K^+^), and hydrogen carbonate (HCO_3_^−^) [48]. It has been reported that salt stress can cause the accumulation of Na^+^ and Cl^−^ while inhibiting the uptake of K^+^ and Ca^2+^, which would perturb osmotic homeostasis. Here we measured the H^+^-antiporter-related gene *NtSOS* and an isoform of high-affinity K^+^ transporter *NtHKT555/586* are critical in improving the salinity tolerance of plants [49,50]. Under stress treatment, the enhanced expression levels of these genes in transgenic tobacco lines showed that *PsnNAC036* might affect the expression of the genes participating in ion exchange to maintain the osmotic homeostasis in plants.

To further understand the regulatory functions of the *PsnNAC036* TF, relative expression levels of 14 stress-related genes (*NtSOD*, *NtPOD*, *NtPPO*, *NtNCED1*, *NtP5CS*, *NtDREB3*, *NtSOS*, *NtLEA5*, *NtERD10A/B/C/D*, and *NtHKT555/586*) were quantified in the *PsnNAC036* transgenic lines and WT under control, salinity, and heat conditions (Figure 7). *NtSOD* and *NtPOD* activities are known to be regulated at their transcription levels [51]. *NtPPO* is a polyphenol oxidase gene involved in stress tolerance in many plants [52]. *NtNECD1* plays an important role in ABA biosynthesis [53]. *NtP5CS* encodes a key enzyme in proline biosynthesis [54]. *NtLEA5* is known to protect osmotic adjustment membrane under stress conditions [55]. *NtDREB3* regulates stress-responsive genes by interacting with specific *cis*-acting elements [56]. *NtERD10A/B/C/D* are associated with dehydration response [54]. The results clearly showed that *PsnNAC036* upregulates these stress responsive genes, leading to enhanced salinity and heat stress tolerance. However, how *PsnNAC036* is activated and regulates the stress-related genes is not known. Investigating how *PsnNAC036* interacts with DNA and proteins would be an exciting future research direction.

## 4. Materials and Methods

### 4.1. Plant Materials and Stress Treatments

*P. simonii × P. nigra* seedlings were grown on 1/2 MS (Murashige and Skoog medium) plant medium (pH5.8), in a growth chamber with a temperature of 25 ± 1 °C, light of 160 μmol/m^2^s and 16/8-h light/dark cycle, and relative humidity of approximately 65%. The one-month-old plants were grown hydroponically and those with new roots and leaves of similar sizes were selected for 150 mM NaCl treatment for 24 h. The control (water) and treated leaves from eight plants (four biological replicates each) were frozen in liquid nitrogen and provided to Beijing GENAWIZ Company (www.genewiz.com accessed on 4 March 2021) for RNA sequencing using Illumina HiSeq2500. RNA-Seq data analysiswas carried out as previously described [18] and the heatmap was generated using MetaboAnalyst (https://www.metaboanalyst.ca accessed on 4 March 2021, Version 5.0). The RNA-Seq data have been deposited in NCBI SRA with the accession number SRP267437.

### 4.2. Cloning and Sequence Analysis of PsnNAC036

Total RNA was extracted from the leaves, and then reversely transcribed into cDNA as a template for PCR amplification. Based on the RNA-Seq analysis, *PsnNAC036* was significantly upregulated in response to high salt treatment. From the PlantTFDB (http://planttfdb.gao-lab.org/family.php?sp=Ptr&fam=NAC accessed on 4 March 2021, Version 5.0), we identified a highly homologous gene of *PsnNAC036* with gene ID Potri.011G123300.1 and designed a pair of primers F1: 5′-ATGGGACTGCAAGAAACAGACC-3′ and R1: 5′-TCACTGCCTAAACCCATACCCA-3′ to clone the *PsnNAC036* gene from *P. simonii × P. nigra*. The amino acid sequence of *PsnNAC036* was used for multi-sequence alignment analysis by BioEdit (Version 7.2). Conserved domain and motifs analyses were conducted using MEME (http://meme-suite.org/tools/meme, Version 5.3.3). Nuclear localization signals (NLS) were predicted with cNLS Mapper (http://nls-mapper.iab.keio.ac.jp/cgi-bin/NLS_Mapper_form.cgi#opennewwindow accessed on 4 March 2021, Version 2012). A phylogenetic tree was constructed using MEGA 7.

### 4.3. PsnNAC036 Gene Expression Analysis

To analyze the relative expression levels of *PsnNAC036* under different stresses, the seedlings were treated with NaCl, ABA, HT, cold, ordrought for 0, 3, 6, 12, 24, and 48 h, respectively. For the salt and ABA treatment, seedlings were incubated in 150 mM NaCl and 50 µM ABA solution, respectively. For drought stress, the seedlings were exposed to air on filter paper for dehydration. For high or low temperature treatment, seedlings were kept in 37 °C or 4 °C illumination incubators. There was only one variable per treatment compared to the control. Leaf and root tissues were harvested with four biological replicatesat each time point for RNA extraction and RT-qPCR. *Actin* (AF: 5′-ACCCTCCAATCCAGACACTG-3′; AR: 5′- TTGCTGACCGTATGAGCAAG-3′) was used as the reference gene in RT-qPCR. The relative expression level in different samples was calculated using 2^−ΔΔCt^ method.

### 4.4. GUS Activity of PsnNAC036 Promoter

DNA was extracted from theleaves using a NuCleanPlantGen DNA Kit (CWBIO, Beijing, China). To clone promoter of *PsnNAC036* from *P. simonii × P. nigra*, the promoter sequence of the *PtrNAC036* gene (https://phytozome.jgi.doe.gov/jbrowse/index.html accessed on 4 March 2021, Populus trichocarpa v3.0)was taken as a reference for designing primers pF: 5′-GCGAAGCTTGTTAGGTGCCGAATCTCCGGTGTCC-3′ and pR: 5′-CGCGGATCCCGGGTGAAACCGAAATCCCGGTGGC-3′, which contain *HindШ* and *BamHI* restriction enzyme sites, respectively (underlined). The promoter sequence was input in PlantCARE (http://bioinformatics.psb.ugent.be/webtools/plantcare/html/ accessed on 4 March 2021) for *cis*-acting element prediction. The promoter sequence was cloned into a pBI121 vector to replace its CaMV35S promoter. Then the recombinant vector was transferred into *Agrobacterium tumefaciens* EHA105. Using a leaf disc method [57,58], it was then transformed into tobacco plants. Three homozygous transgenic lines P1, P2, and P3 and WT (control) were treated with 150 mM NaCl, 50 µM ABA, HT (37 °C), cold (4 °C), or drought for 12 h, respectively. Histochemical assays of *GUS* enzyme activity driven by the *PsnNAC036* promoter were conducted as previously described [59].

### 4.5. Subcellular Localization of the PsnNAC036 Protein

The *PsnNAC036* open reading frame (ORF) lacking the stop codon was cloned using primers F3:5′-GGGTCGACTGACTAGTATGGGACTGCAAGAAACAGACC-3′, and R3: TGCTCACCATACTAGTCTGCCTAAACCCATACCCACTT-3′, which contain a *SpeI* restriction site (underlined).Then the sequence was ligated into the linearized pBI121 vector that contains a green fluorescent protein (GFP)using an In-Fusion HD Cloning Kit (TaKaRa, China). The fusion plasmid *35S*::*PsnNAC036*-*GFP* and the control plasmid *35S*::*GFP* were introduced into onion epidermis according to a published method [14]. The GFP fluorescence was observed using a confocal laser scanning microscope (LSM 700, Zeiss, Germany). The results were reproduced three times, and each one had 10 biological replicates.

### 4.6. Transcriptional Activation Assay of the PsnNAC036 Protein

The ORF of *PsnNAC036* (1–343amino acids (aa)) and the different truncations of the *PsnNAC036* encoding the N-terminal NAM domain 1–139 aa, non-conserved domain 140–343 aa, and the short ORF fragments 140–241 aa, 242–343 aa, 140–190 aa, 191–241 aa, 242–292 aa, and 293–343 aa were inserted into the pGBKT7 vector, respectively. These constructs were named as *BD-NAC036*, *BD-NAC036a*, *BD-NAC036b*, *BD-NAC036c*, *BD-NAC036d*, *BD-NAC036e*, *BD-NAC036f*, *BD-NAC036g*, and *BD-NAC036h*, respectively (Figure 3). The negative control *pGBKT7* plasmid (*BD*) and all the constructs were transformed into Y2H yeast strain. Transformants were grown for 3–5 days on a selective medium without *Trp* and *His*. *β*-Galactosidase assay was performed on filter lifts of the colonies to detect activation of the *lacZ* reporter gene.

### 4.7. PsnNAC036 Overexpression Vector Construction for Plant Transformation

On the basis of the *PsnNAC036* sequence, we designed a pair of primers F4: 5′-GCGTCTAGAATGGGACTGCAAGAAACAGACC-3′ and R4: 5′-GCGGAGCTCTGGGTATGGGTTTAGGCAGTGA-3′, which contain *XbaI* and *SacI* restriction sites, respectively (underlined). Both the amplified fragment and the plant binary expression vector pBI121 were double digested by *XbaI* and *SacI* and then were ligated together by T4 ligase. The recombinant plasmid *PsnNAC03*6-pBI121 was confirmed by PCR using the vector primers pBI121F1:5′-CCATCGTTGAAGATGCCTCTGC-3′ and pBI121R1:5′-CTCTTCGCTATTACGCCAGCTGG-3′. Positive clones were transferred into *Agrobacterium tumefaciens* EHA105 for plant genetic transformation.

### 4.8. Generation of PsnNAC036 Transgenic Tobacco Plants

One-month-old tobacco seedlings were used for *PsnNAC036* transformation by a leaf disc method [2,19]. *A.tumefaciens* EHA105 containing the recombinant vector *PsnNAC036*-*pBI121* were cultured in LB liquid medium with rifampicin (50 mg/mL) and kanamycin (50 mg/mL) until OD_600_ = 0.6. Tobacco leaves were cut into 1 × 1 cm squares and incubated in the bacterial solution for 15 min.The leaves were then cultured in MS media with kanamycin (100 mg/mL) for selection. Total RNA of each line wasextracted for PCR with the specific primers F1 and R1. WT was regarded as a negative control and the *PsnNAC036-pBI121* plasmid as a positive control (P). Three independent homozygous transgenic lines T1, T2, and T3 were selected for subsequent experiments.

### 4.9. Root Length of the PsnNAC036 Transgenic Tobacco

To determine salinity and HT tolerance of thetransgenic tobacco overexpressing *PsnNAC036*, seeds of the transgenic lines T1, T2, T3, and WT were grown on the MS medium for seven days. Then they were transplanted into MS containing 150 mM NaCl (salt treatment) or grown in a 37 °C (HT treatment) illumination incubator. Root length was measured two weeks after the transplant.

### 4.10. Chlorophyll Content and Physiological measurement

Chlorophyll a and chlorophyll b were extracted and quantified from leaves of the transgenic lines and WT plants after salt and HT treatments following a previous method [60]. Total chlorophyll content was calculated according to the following equations: Chlorophyll a = [(12.72 × A_663_ − 2.69 × A_645_)V/W], Chlorophyll b = [(22.88 × A_645_ − 4.68 × A_663_)V/W], total chlorophyll content = Chlorophyll a +Chlorophyll b, where A_663_ and A_645_ are absorbance at 663 nm and 645 nm, respectively. V is the final volume of chlorophyll extract in 80% acetone, and W is fresh weight of leaves. Biochemical analyses of superoxide dismutase (SOD), peroxidase (POD), proline content, and malondialdehyde (MDA) were conducted according to previous methods [14].

### 4.11. Histochemical Analyses of the PsnNAC036 Transgenic Tobacco

Cell death, accumulation of hydrogen peroxide (H_2_O_2_) and superoxide (O_2_^−^) in the transgenic and WT plants under high saltor HT were visualized through histochemical staining with Evans blue, 3,3′-diaminobenzidine (DAB) and nitro-blue tetrazolium (NBT), respectively. Two-week-old seedlings were irrigated with water (control), 150 mM NaCl solution or grown in 37 °C HT for 12 h. Leaves were excised for histochemical staining as previously described [10].

### 4.12. Salt and HT Treatments of the PsnNAC036 Transgenic Tobacco

The seedlings of WT and transgenic tobacco lines on MS medium were transplanted in the soil oncethe fourth leaf appeared. The seedlings were grown under normal growth conditions for a total of two weeks, and then were subjected to 200 mM NaCl irrigation or 37 °C HT treatment for another two weeks.

### 4.13. Expression Analysis of Stress-Related Genes in PsnNAC036 Transgenic Tobacco

To further understand the functions the *PsnNAC036* TF, leaves of the transgenic lines and WT under 200 mMNaCl or 37 °C HT for 12 h were used for RT-qPCR. Relative expression levels ofstress-related genes including superoxide dismutase (*NtSOD*), peroxidase (*NtPOD*), polyphenol oxidase (*NtPPO*), 9-cis-epoxycarotenoid dioxygenase1 (*NtNCED1*), 1-pyrroline-5-carhoxylate synthetase (*NtP5CS*), regulatory proteins (*NtDREB3*), plasmalemma Na^+^/H^+^ antiporter (*NtSOS*), late-embryogenesis-abundant protein5 (*NtLEA5*), early responsive to dehydration (*NtERD10A/B/C/D*) and Na^+^ antiporter genes (*NtHKT555/586*) were quantified. RT-qPCR was conducted according to a published method [14]. The primer sequences used for the above genes can be found in Supplementary Table S2.

## 5. Conclusions

In this study, we analyzed the expression changes of NAC TFs in *P. simonii × P. nigra* under 150 mM salt treatment and isolated a significantly upregulated NAC gene, *PsnNAC036*. RT-qPCR and promoter *GUS* analyses showed that the gene was highly responsive to stresses, especially salinity and heat. Overexpression of *PsnNAC036* improved salinity and HT tolerance of transgenic plants. RT-qPCR results revealed that overexpression of the *PsnNAC036* TF regulated the expression of several stress-related genes in the transgenic plants. Furthermore, we validated that the C-terminal domain (191–343 amino acids) was the potent activator of *PsnNAC036*. These results have demonstrated that *PsnNAC036* functions as a transcriptional activatorand plays an important role in plant salinity and HT tolerance. As such, it is a potential molecular target for crop biotechnology and marker-based breeding for enhanced resilience and yield.

## Supplementary Materials

Supplementary materials can be found at https://www.mdpi.com/1422-0067/22/5/2656/s1.
