# Supplementary material for: Functional Characterization of PsnNAC036 under Salinity and High Temperature Stresses
Source: Int J Mol Sci. 2021 Mar 6;22(5):2656. doi: 10.3390/ijms22052656 (PMC7961394; doi:10.3390/ijms22052656)
Supplement: Supplementary file 1 [file ijms-22-02656-s001.pdf]

Supplementary Figures

Supplementary Figure S1

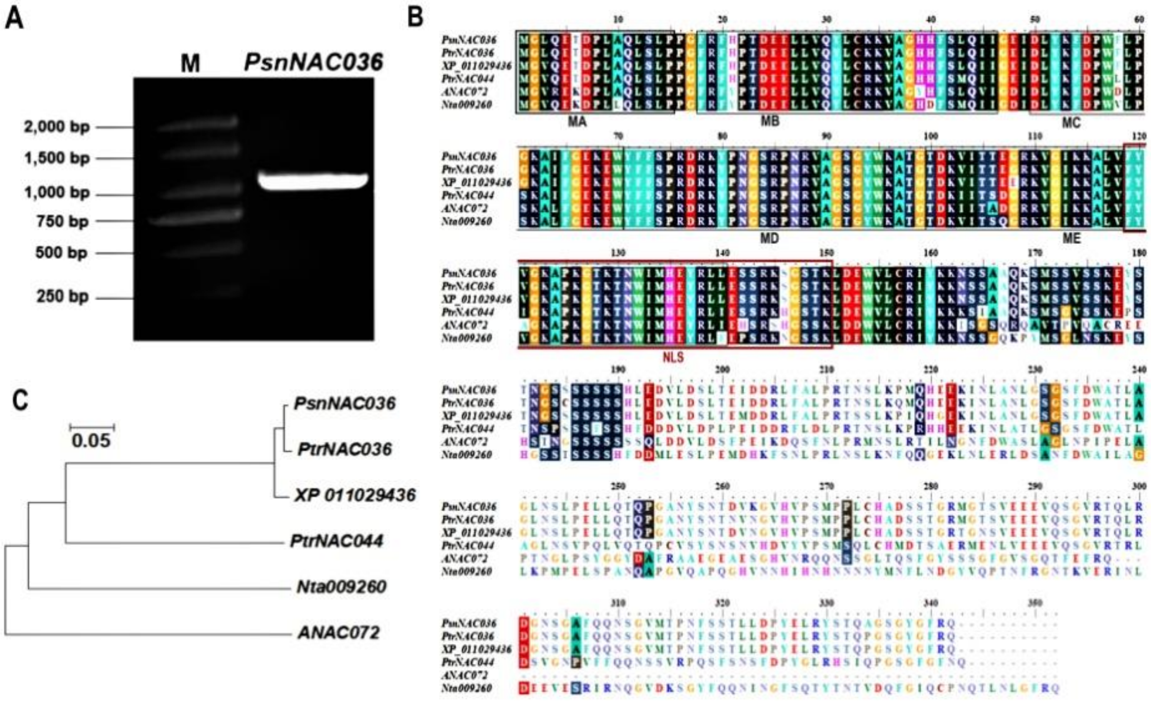

Supplementary Figure 1**.** Bioinformatics analysis of the *PsnNAC036* TF. **(A)** Cloning of ORF of *PsnNAC036* from *P. simonii* × *P. nigra*. **(B)** Amino acid sequence alignment of *Psn*NAC036 with homologous proteins from other species. The five NAC motifs MA, MB, MC, MD and ME forming the highly conserved NAM domain were marked. The predicted nuclear localization signal (NLS) sequence was shown in a red box. **(C)** Phylogenetic tree analysis of *PsnNAC036* by MEGA7 with the Neighbor Joining method.

Supplementary Figure S2

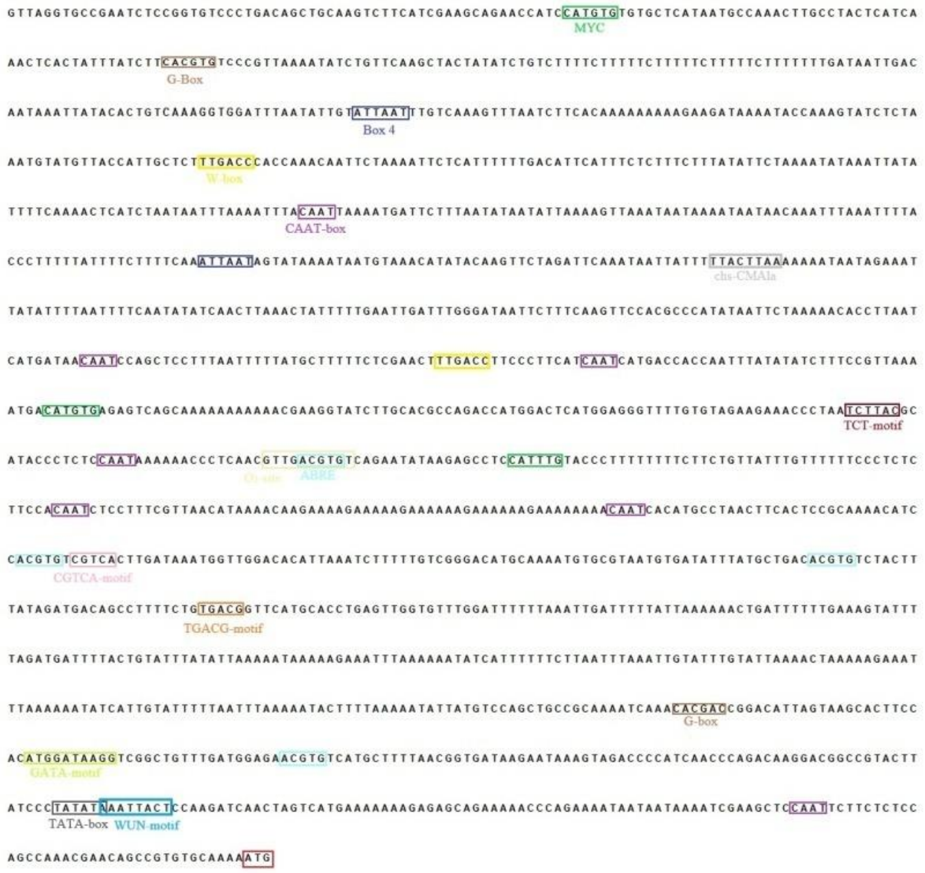

Supplementary Figure 2. Sequence of the *PsnNAC036* promoter and various *cis*-elements marked with different colored boxes.

Supplementary Figure S3

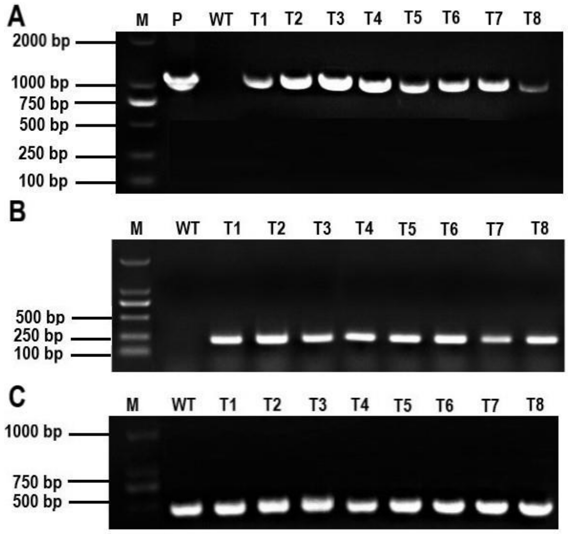

Supplementary Figure 3. Validation of overexpression transgenic lines. (A) gDNA PCR detection with specific primers F1 and R1. (B) Dection with primers F2 and R2. (C) Dection with reference primers *actin*. M, 2000 DNA marker; P, positive plasmid; WT, wild type.

Supplementary Figure S4

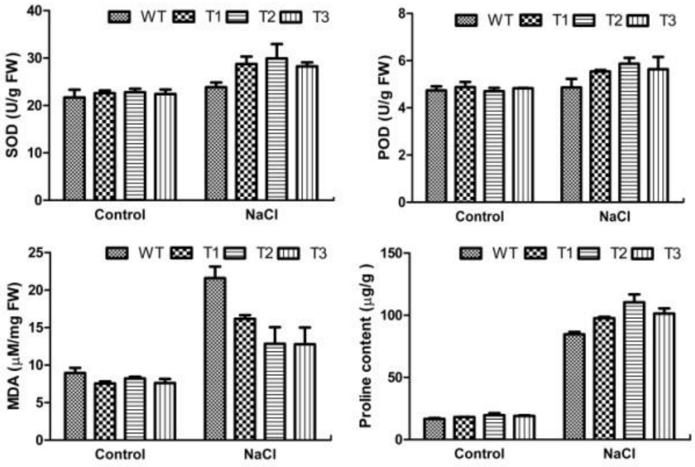

Supplementary Figure 4. Plysiological analysis of WT and transgenic tobacco lines. WT, wild type; T1-T3: overexpressing transgenic tobacco lines.

Supplementary Figure S5

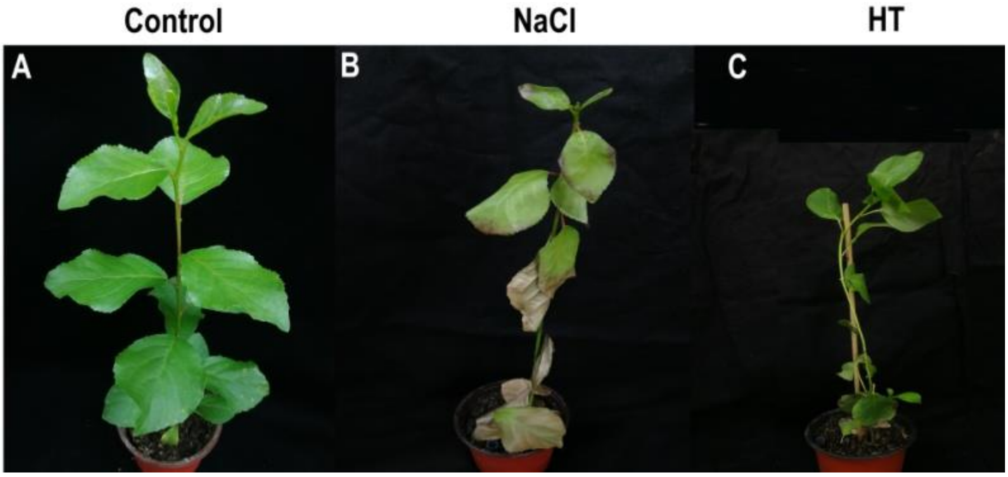

Supplementary Figure 5. Phenotype of one-month-old *P. simonii* × *P. nigra* under NaCl and HT treatments.**(A)** WT poplar under control condition; **(B)** WT poplar under 150 mM NaCl treatment for two weeks; **(C)** WT poplar under 37℃ for two weeks.

Supplementary tables

Supplementary Table S1 Promoter elements of *PsnNAC036* gene

Element	Sequence	Function
AAGAA-motif	GAAAGAA	involved in seed specific expression
AT~TATA-box	TATATAAA/TATATA	efficiencymotifs of mRNA 3′-end formation
CAAT-box	CAAT/CAAAT	*cis*-acting element and enhancer region
AT-rich element	ATAGAAATCAA	binding site of AT-rich DNA binding protein
chs-CMA2a	TCACTTGA	light responsive element
A-box	CCGTCC	*cis*-acting regulatory element
ERE	ATTTTAAA	ethylene-responsive element
Box 4	ATTAAT	part of a conserved DNA module involved in light responsiveness
I-box	GGATAAGGTG	part of a light responsive element
O2-site	GTTGACGTGA	involved in zein metabolism regulation
W box	TTGACC	WRKY plant specific zinc-finger-type factor associated with pathogen defense
WUN-motif	AAATTTCTT/AAATTACT	wound-responsive element
GATA-motif	AAGGATAAGG	part of a light responsive element
LAMP-element	CCTTATCCA	part of a light responsive element
CGTCA-motif	CGTCA	involved in the MeJA-responsiveness
G-box	CACGTG/CACGTC/GCCACGTGGA	light responsiveness and combines with other regulatory elements under specific stress
TATA-box	TATA	core promoter element around -30 of transcription start and important for recognition by RNA polymerase Ⅱ

AT-rich sequence	TAAAATACT	element for maximal elicitor-mediated activation (2copies)
TCT-motif	TCTTAC	part of a light responsive element
MYC	CATGTG/CATTTG	involved in chilling response
MYB	CAACCA	MYB binding site
TATC-box	TATCCCA	*cis*-acting element involved in gibberellin-responsiveness
ABRE	CACGTG/ACGTG/GACACGTGGC	involved in the abscisic acid responsiveness
chs-CMA1a	TTACTTAA	part of a light responsive element
as-1	TGACG	oxidative stress-responsive element
DRE core	GCCGAC	dehydration responsive element
TGACG-motif	TGACG	element involved in the MeJA-responsiveness

Supplementary Table S2 Primers of stress-related genes

Gene name	Gene ID	Forward primer (5'-3')	Reverse primer (5'-3')
Ntactin	U60489	CATTGGCGCTGAGAGATTC	GCAGCTTCCATTCCGATCA
NtUbiquitin	U66264.1	AAAGAGTCAACCCGTCACCT	ACATCACGACCACAACCAGA
NtSOD	AB093097	CGGCAATTAGCGGTGACATA	ATGGCGTCATGTAGCTGTTC
NtPOD	AB178953	CTCCATTTCCATGACTGCTTTG	GTTGGGTGGTGAGGTCTTT
NtPPO	A27686.1	AACCCGTTCCGTGTGAAAGTCC	CTTCGATTACGCACCGATGCCA
NtSOS	LOC107768444	TCCCAAAGAATAGGTGCC	TGGATGACGAAGAACCACT

NtNCED1	HM068892	ACGAACTCCAACACCCTTTAC	AGGGAGTGAGAGACTGGATTT
NtP5CS	HM854026	GACACGGACTGATGGAAGATTAG	GCACCTGAAGTCACCAGAATAA
NtDERB3	EU727157	GCCGGAATACACAGGAGAAG	CCAATTTGGGAACACTGAGG
NtLEA5	AF053076	GTTACCATACCACGTCCCATAG	GAGCTAGGACGCTCCATATTT
NtERD10A	AB049335	TCTGAAGCGTGGCACTATTT	TCCACGGCACATCACTATAAC
NtERD10B	AB049336	CAACTGCAACAACTACGACT	GGTGGCCAGGAAGCTTCT
NtERD10C	AB049337	AACGTGGAGGCTACAGATCG	GTTCCTCTTGGGCATGAGTT
NtERD10D	AB049338	GAGGACACGGCTGTACCAGT	GCGCCACTTCCTCTGTCTT
NtHKT555	LOC107787555	AACCTCCACCTTCGCTATT	GAACCCAAACACCGTAACC
NtHKT586	LOC107781586	GCCTCCACAAATCCATTC	TGCTTGAGACAGTTACCGAA

Ntactin and *NtUbiquitin* were reference genes for RT-qPCR.
